# A Comparison of Doctor/Patient Satisfaction with Aesthetic Outcomes of Rhinoplasty: a Prospective Study

**DOI:** 10.25122/jml-2019-0061

**Published:** 2019

**Authors:** Fatemeh Rezaei, Farzad Rezaei, Hoshyar Abbasi, Hamed Moradi

**Affiliations:** 1. Department of Oral Medicine, School of Dentistry, Kermanshah University of Medical Sciences, Kermanshah, Iran; 2. Department of Oral and Maxillofacial Surgery, School of Dentistry, Kermanshah University of Medical Sciences, Kermanshah, Iran; 3. Department of Oral and Maxillofacial Surgery, School of Dentistry, Kermanshah University of Medical Sciences, Kermanshah, Iran; 4. School of Dentistry, Kermanshah University of Medical Sciences, Kermanshah, Iran

**Keywords:** rhinoplasty, aesthetics, patient satisfaction, nose job, quality of life

## Abstract

The tendency toward cosmetic surgeries has been increasing all over the world. These surgeries are conducted to improve the functions related to the involved organ. Moreover, such procedures are supposed to have significant effects on an individual’s physical and mental health. This study aims at comparing doctor/patient satisfaction with the aesthetic outcomes of rhinoplasty.

In the present retrospective study, 60 patients, including 26 males and 34 females, were investigated. The patients were candidates for a nose job (rhinoplasty). One week before the surgery, the doctor and the patients were provided with questionnaires including information about the nasal hump, nostrils, nose tip size, upward sloping of the nose at the tip, the display of the middle-lower nose, nasal width, the nasal proportion compared with the face, and the general satisfaction. The information on the aforementioned items was collected to record the condition of nose beauty before rhinoplasty. After the surgery, the patients and the doctor were given the same questionnaire following one-month and three-month intervals, so that the aesthetic outcomes resulted from the surgery were evaluated.

In comparison to the first month, the surgeons and the patients had a higher level of satisfaction in the third month. The surgeons’ satisfaction with the upward sloping of the nose at the tip, as well as the display of the middle-lower nose, was greater compared to the patients. However, the patients’ satisfaction with the nostrils was higher than that of the surgeons. Regarding the other factors investigated in the present study, there was no significant difference between the satisfaction of patients and surgeons. For individuals whose educational levels were higher than high school, there was a higher satisfaction level with the nasal hump, the display of the middle-lower nose, and the nasal proportion compared with the face.

## Introduction

Rhinoplasty is one of the most common types of cosmetic surgeries conducted by plastic surgeons. In comparison to other cosmetic surgeries, rhinoplasty is commonly associated with a lower level of patient satisfaction [1]. Rhinoplasty is a surgery in which the appearance and performance of the nasal structures such as nose skin, cartilage, and bones undergo some changes. This kind of surgery is divided into two types: open and closed. Even for surgeons that perform this nearly every day, rhinoplasty is one of the hardest types of plastic surgery. The complications of rhinoplasty are widely discussed in scientific seminars and assemblies as a widespread and significant field of surgery [2]. Nowadays, rhinoplasty is considered the best-known method for improving and reshaping the nose. Rhinoplasty is divided into two types. Type 1 is called corrective: it refers to cases in which the patient’s nose is not acceptable in terms of shape and performance. Type 2 is conducted for cosmetic purposes; the main aim is changing the appearance of the nose and maintaining its performance [3, 4].

There are different methods for conducting rhinoplasty. Adopting the proper method depends on the patient’s nose shape and his/her demands and expectations from the intended surgery regarding his/her nose form and size. In fact, it can be claimed that there is no standard rhinoplasty; the existing methods vary widely, and depend on the type of nose deformity [5)].

The studies have indicated that the main factors of willingness for cosmetic surgery include dissatisfaction with one’s appearance, willingness to have an ideal appearance, gender, partner, high income and social level, and high educational level [6].

It is of high significance to evaluate the complications of cosmetic surgeries by measuring the patients’ satisfaction with their quality of life. Thus, for evaluating the quality of life as well as self-knowledge, questionnaires are regarded as standard tools for collecting information and making objective comparisons of processes through measuring post-rhinoplasty positive and negative effects; questionnaires are significantly useful in evaluating the success of facial cosmetic surgeries [7-9].

Patients’ satisfaction depends on subjective factors, including awareness of pre-rhinoplasty appearance, expectations, the capability of establishing social relationships, alcohol use, and mood [10]. According to patients and surgeons, the most important aesthetic concerns are asymmetry, tip sloping of the middle one-third of the nose, and deformity of the upper one-third [9].

According to the findings of the study conducted by Grossbart et al., cosmetic surgery has positive effects on the patients’ behaviors, mood, and self-esteem [11]. Moreover, cosmetic surgery is considered a useful step toward improving the quality of life in these individuals.

One of the main advantages of conducting the present study is investigating the positive and negative aspects of rhinoplasty on the physical and mental health based on the patients’ age, gender, educational level, and marital status. It is also attempted to identify groups whose satisfaction and quality of life were significantly affected by rhinoplasty. Moreover, the findings of the present study help surgeons predict the patients’ satisfaction so that the patient’s pre-rhinoplasty aesthetic views are better understood. Thus, the patients’ post-rhinoplasty satisfaction increases as well.

In the present study, it was attempted to analyze the views of both patients and surgeons. Moreover, it was attempted to investigate the relationship between patients’ age, gender, educational level, and marital status with factors including nasal hump, nostrils, nose tip size, upward sloping of nose at the tip, the display of the middle-lower nose, nasal width, proportion of the nose compared with the face, and the general satisfaction. This comparison was conducted one month and three months after rhinoplasty.

## Material and Methods

In the present study, some patients candidates for open rhinoplasty treatment were selected. The inclusion criteria were as follows:

• Lack of any congenital deformity of the jaw or face;
• Age range of 18-35 years;
• Lack of any pathologic lesions and deformities of the jaw or face;
• Lack of a previous rhinoplasty;
• Lack of systemic problems;
• Lack of history of psychological problems.

In the present study, it was attempted to analyze the views of both patients and surgeons. Moreover, it was attempted to investigate the relationship between patients’ age, gender, educational level, and marital status with factors including nasal hump, nostrils, nose tip size, upward sloping of nose at the tip, the display of the middle-lower nose, nasal width, proportion of nose with face, and the general satisfaction. This comparison was conducted one month and three months after rhinoplasty.

One week before rhinoplasty, the patients and the surgeon were provided with a questionnaire to record the status of nose beauty.

In this questionnaire, there were questions regarding the patient’s demographic information. Moreover, there were questions about the patients’ satisfaction with his/her face and nose. Before completing the questionnaire, the patients were provided with the required information regarding the questionnaire and purpose of the intended study.

The questionnaire was researcher-made. The Kappa index was applied for measuring its reliability and validity. One week after receiving the phase-two questionnaire (one month after the surgery), the patients and the doctor were once more provided with the questionnaire.

### Ethical considerations

The patients entered the present study with complete awareness of the phases and purposes of the research. Moreover, written letters of consent were acquired from the patients. They were also allowed to leave the study at any stage of the study willingly. The information obtained from the patients were kept confidential. The present study was confirmed by the Ethics Committee of Kermanshah University of Medical Sciences.

### Data collection and sample size

After collecting the intended questionnaire that included demographic information and variables related to nasal satisfaction and beauty, the information was entered into SPSS and analyzed by applying statistical analyses. For analyzing the reliability of the questionnaire, one week after receiving the phase-two questionnaire (one month after the surgery), the patients and the doctor were once more provided with the questionnaire. Then, the Kappa index was applied for measuring its reliability and validity.

For measuring the sample size, it was attempted to apply the findings of the previous studies. In the study conducted by Sena Esteves et al., the variable standard deviation of the rhinoplasty outcomes evaluation (ROE) was 51.82σδ2. By considering the accuacy of d=7, α=0.05, and the power 1-ß=90%, the minimum sample size was measured to be 50 individuals. For having more certainty, the questionnaires were completed by 60 participants; the sample size was measured through the following formula:


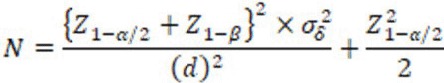


### Statistical analysis

The analysis of the data collected in the present study was conducted in two parts, i.e., descriptive statistics and inferential statistics. Regarding the descriptive statistics, measures of the central tendency and distribution have been reported in tables. As for the inferential statistics, the Chi-squared test, Fisher’s exact test, Monte Carlo chi-square test, and Wilcoxon signed-rank test were used.

For analyzing the data, SPSS 18.0 (Inc., Chicago, IL, USA) and R Version 3.2.2 with the “dunn.test” package were applied. The significance level of the present study was designed to be 0.05.

## Results

There is a significant correlation between age, gender, marital status, and educational level with the patient’s general satisfaction level.

• The comparison of patient satisfaction (at one month and three-month intervals after rhinoplasty): There is a significant relationship between general satisfaction and patient satisfaction with the nasal hump, upward sloping of the nose, and the display of the middle-lower nose.
• The comparison of surgeon’s satisfaction (at one month and three-month intervals after rhinoplasty): There is a significant relationship between the surgeon’s satisfaction (at one month and three-month intervals after rhinoplasty) and the general satisfaction with one’s nose.
• The comparison of patient-surgeon satisfaction (one month after rhinoplasty): There is a significant relationship between patient-surgeon satisfaction (one month after rhinoplasty) and satisfaction with nostrils, upward sloping of the nose, and the display of the middle-lower nose.
• The comparison of patient-surgeon satisfaction (three months after rhinoplasty): There is a significant relationship between the display of the middle-lower nose and general satisfaction.

All results are shown in Tables 1-9.

**Table 1:** The effect of patient’s gender on his/her satisfaction with the nostrils (one month after rhinoplasty).

Factor	Satisfaction level	Patient’s gender			
		Male		Female	
		Number	Percentage	Number	Percentage
**Nostrils (one month after rhinoplasty)**	**Completely satisfied**	4	17	11	29
	**Satisfied**	9	39	15	40
	**Relatively satisfied**	7	30	10	27
	**Dissatisfied**	3	13	1	2

**Table 2:** The effect of patient’s age on his/her satisfaction with different factors (one month and three months after.

Factor	Satisfaction level	Patient’s age			
		Younger than 25 years		Older than 25 years	
		Number	Percentage	Number	Percentage
**Nasal width (one month after rhinoplasty)**	**Completely satisfied**	18	56	9	32
	**Satisfied**	13	40	17	60
	**Relatively satisfied**	1	3	1	3
	**Dissatisfied**	0	0	1	3
**Display of the middle-lower nose (three months after rhinoplasty)**	**Completely satisfied**	26	81	18	64
	**Satisfied**	4	12	3	10
	**Relatively satisfied**	2	6	5	17
	**Dissatisfied**	0	0	2	7
**The general satisfaction level (three months after rhinoplasty**	**Completely satisfied**	16	50	9	32
	**Satisfied**	15	46	41	50
	**Relatively satisfied**	1	31	4	14
	**Dissatisfied**	0	0	1	3

**Table 3:** The effect of patient’s marital status on his/her satisfaction with the display of the middle-lower nose (three months after rhinoplasty).

**Factor**	**Satisfaction level**	**Marital status**			
		**Single**		**Married**	
		Number	Percentage	Number	Percentage
**Display of the middle-lower nose (three months after rhinoplasty)**	**Completely satisfied**	32	80	12	60
	**Satisfied**	4	10	15	3
	**Relatively satisfied**	4	10	3	15
	**Dissatisfied**	0	0	2	10

**Table 4:** The effect of educational level on his/her satisfaction with different factors (one month after rhinoplasty).

Factor	Satisfaction level	Educational level				P-value
		Lower than high school diploma	Higher than high school diploma			
		Number	Percentage	Number	Percentage	
**Nasal hump (one month after rhinoplasty)**	**Completely satisfied**	5	21	4	17	0.009
	**Satisfied**	6	26	9	39	
	**Relatively satisfied**	9	39	6	26	
	**Dissatisfied**	4	17	4	17	
**Display of middle-lower nose**	**Completely satisfied**	5	21	21	56	0.003
	**satisfied**	12	52	13	35	
	**Relatively satisfied**	2	8	2	5	
	**Dissatisfied**	4	17	1	2	
**Nose proportions (one month after rhinoplasty)**	**Completely satisfied**	5	26	21	56	0.002
	**satisfied**	13	56	16	43	
	**Relatively satisfied**	3	13	0	0	
	**Dissatisfied**	1	4	0	0	
**General satisfaction (one month after rhinoplasty)**	**Completely satisfied**	4	17	11	29	0.014
	**satisfied**	14	60	26	70	
	**Relatively satisfied**	4	17	0	0	
	**Dissatisfied**	1	4	0	0	

**Table5:** The effect of the patient’s educational level on his/her satisfaction with the nasal hump (three months after rhinoplasty).

Factor	Satisfaction level	Educational level			
		Lower than high school diploma		Higher than high school diploma	
		Number	Percentage	Number	Percentage
**Nasal hump (three months after rhinoplasty)**	**Completely satisfied**	10	43	21	56
	**Satisfied**	3	13	10	27
	**Relatively satisfied**	7	30	6	16
	**Dissatisfied**	3	13	0	0

**Table 6:** The comparison of surgeons’ opinions one month and three months after rhinoplasty.

Factor	The comparison of surgeons’ opinions		P-value
**Satisfaction level**	**One month after rhinoplasty**	**Three months after rhinoplasty**	0.000
	**Percentage**	**Percentage**	
	33	61	

**Table 7:** The comparison of patients’ opinions one month and three months after rhinoplasty.

Factor	The comparison of patients’ opinions		P-value
Interval	One month after rhinoplasty	Three months after rhinoplasty	
Satisfaction level	Percentage	Percentage	
**Nasal hump**	25	41	0.003
**Upward sloping of the nose**	38	61	0.001
**Nose proportions**	43	73	0.043

**Table 8:** The satisfaction opinions of patients and surgeons one month after rhinoplasty.

Factor	The satisfaction opinions of patients and surgeons (one month after rhunoplasty)		P-value
Satisfaction level	Percentage	Percentage	
**Nostrils**	25	45	0.043
**Upward sloping of nose**	56	43	0.006
**Display of middle-lower nose**	65	43	0.000

**Table 9:** The satisfaction opinions of patients and surgeons three months after rhinoplasty.

Satisfaction level	The comparison between patients and surgeons’s opinions three month after rhinoplasty		P-value
	Surgeons	Patients	
	Percentage	Percentage	
**Display of middle-lower nose**	78	73	0.042
**General satisfaction**	61	41	0.009

There is a significant statistical difference in patients’ satisfaction with the nostrils (one month after rhinoplasty) in terms of the patient’s gender (p-value: 0.031); women’s satisfaction level is higher than that of men’s.

As shown in Table 2, in terms of patients’ satisfaction with nasal width (one month after rhinoplasty), the P-value (0.042) indicated that the satisfaction level of patients older than 25 years was higher. In terms of the patients’ satisfaction with the display of the middle-lower nose (three months after rhinoplasty), the P-value was 0.032; this indicates that the satisfaction level of patients younger than 25 was higher. In terms of the patients’ general satisfaction level (three months after rhinoplasty), the P-value of 0.033 indicated that the satisfaction level of individuals younger than 25 was higher.

There is a significant statistical difference between married participants and single ones regarding the patients’ satisfaction in terms of the middle-lower nose display (three months after rhinoplasty) (P-value: 0.038); the satisfaction of single participants was higher compared to married participants.

Given the data and p-values, the satisfaction with the nasal hump (three months after rhinoplasty) was higher in patients that did not have a high school diploma. The satisfaction with the display of a middle-lower nose (three months after rhinoplasty) was higher among the patients having a higher education level. Regarding the satisfaction with the proportion of the nose compared to the face (one month after rhinoplasty), there was a higher level of satisfaction among the patients having a higher educational level. In terms of the patients’ general satisfaction (one month after rhinoplasty), there was a higher level of satisfaction among the patients having a higher educational level (more than a high school diploma).

There is a significant statistical difference regarding the patients’ satisfaction with the nasal hump between patients having a high educational level and those having just a high school diploma (P-value: 0.023); the satisfaction level was higher among the patients with a high level of education.

There is a significant statistical difference regarding the surgeons’ opinions about the nasal hump one month and three months after rhinoplasty; their satisfaction level was higher three months after rhinoplasty.

The comparison regarding the patients’ satisfaction with the nasal hump one month and three months after rhinoplasty indicated that their satisfaction level was higher three months after rhinoplasty.

There is a significant statistical difference in the satisfaction of patients and surgeons regarding nostrils, upward sloping of the nose, and the display of the middle-lower nose one month after rhinoplasty. The patients had a higher level of satisfaction with the upward-sloping of the nose and display of the middle-lower nose. However, the surgeons were more satisfied with the nostrils.

Given the data shown in the above tables, it can be concluded that the surgeons had a higher level of satisfaction with the display of the middle-lower nose compared to the patients. Moreover, in comparison to the patients, the surgeons had a higher general satisfaction as well.

## Discussion

Given the individual anatomy, the facial area, especially the nose, is of a higher significance in one’s beauty. Thus, conducting nasal cosmetic surgeries (including rhinoplasty) can significantly affect the individual’s beauty and facial proportion. Rhinoplasty is regarded as one of the most common types of cosmetic surgery conducted by plastic surgeons. In comparison to other types of cosmetic surgeries, rhinoplasty is associated with a lower level of satisfaction [11]. In comparison to primary rhinoplasty, conducting a revision rhinoplasty is more challenging, because its primary purpose is removing the functional or static defects after a failed previous surgery so that the patients’ expectations are fulfilled [12].

The complications of every surgery can be measured through qualitative and quantitative terms. In plastic surgery cases, the surgical processes are commonly optional, and they are conducted for aesthetic purposes. There is no standard quality criterion. Thus, it is hard to provide an objective comparison of the success of the surgeons’ different techniques and individual skills [13]. The personal reports of complications are regarded as an important tool in evaluating the effectiveness of the known medical processes. Thus, the questionnaires were created for evaluating the quality of life and self-knowledge. The questionnaires are applied as standards for collecting the required information and providing the possibility of conducting an objective comparison of surgical processes through measuring post-rhinoplasty positive and negative effects. Thus, questionnaires are significantly helpful for evaluating the success of facial cosmetic surgeries [8].

Factors including age, gender, educational level, and marital status can affect the patients’ expectations and satisfaction with rhinoplasty [14]. Moreover, other individual factors can affect nasal beauty and proportions; these factors are significantly taken into account by patients as success criteria of rhinoplasty (15). On the other hand, patients and surgeons are likely to have different views regarding post-rhinoplasty nasal beauty and proportion. In most cases, the patients and surgeons have similar ideas regarding the beauty and proportion of the conducted rhinoplasty. According to patients and surgeons, the most important aesthetic concerns include

1. Asymmetry;
2. Upward sloping of the middle one-third of the nose;
3. Deformity of the upper one-third.

Nearly 79% of the concerns reported by patients have been reported by the surgeons as well [14].

In the present study, it was attempted to investigate the relationship between the patients’ age, gender, educational level, and marital status with factors including nasal hump, nostrils, nose tip size, upward sloping of the nose at the tip, the display of the middle-lower nose, nasal width, proportion of the nose compared with the face, and the general satisfaction. The relationship of these parameters was not significant i.e., had no effect on one another. This comparison was conducted one month and three months after rhinoplasty.

Salah et al. have investigated post-rhinoplasty satisfaction and quality of life. The findings of their study indicated that the patients’ post-rhinoplasty satisfaction is significantly high in patients [6]. Similarly, in the present study, the patients’ post-rhinoplasty satisfaction is significantly higher. In comparison to the first month’s satisfaction, the satisfaction level with rhinoplasty is higher in the third month.

Yu et al. have evaluated the patients’ functional and static concerns after a revision rhinoplasty and compared them with the objective deformities found by the surgeons. The most important concerns of the patients include

1. Asymmetry;
2. The respiratory problems of the tip or nasal obstruction;
3. The sloping of the middle one-third of the nose [14].

Swami et al. have investigated the factors affecting satisfaction with rhinoplasty. The findings of their study indicated that the tendency towards cosmetic surgeries is positively and significantly associated with the individuals’ cultural-social attitudes about appearance, and this tendency is negatively associated with satisfaction with one’s appearance, age, and body mass index. The effect of media, as well as the lack of satisfaction with one’s appearance and body mass index, significantly increase the tendency towards cosmetic surgeries [16]. Similarly, in the present study, the patients’ age was significantly associated with satisfaction with one’s nasal width one month after rhinoplasty, the display of middle-lower nose three months after rhinoplasty, and general satisfaction three months after rhinoplasty. Individuals younger than 25 years old had a higher level of satisfaction in terms of these three factors (nasal width, display of the middle-lower nose, and general satisfaction) in comparison to those who were older than 25 years.

Litner et al. have investigated the effects of cosmetic facial surgery on satisfaction with appearance and quality of life. The findings of their study indicated that the quality of life of the patients promoted to a significant level. Men and women seem to have different needs in terms of cosmetic surgeries; these surgeries affect their quality of life in different ways [17]. Similarly, in the present study, the patient’s gender affected their satisfaction level in terms of nostrils; the satisfaction level with the nostrils one month after rhinoplasty was higher in women (29%) than that of men (17%).

The patients’ marital status had a significant relationship with the display of the middle-lower nose three months after rhinoplasty; the single patients had a higher level of satisfaction compared to the married ones.

The patients’ educational level had a significant relationship with the nasal hump, the display of the middle-lower nose, the proportion of the nose compared to the face, and the general satisfaction one month after rhinoplasty. Moreover, the educational level had a significant level with the nasal hump three months after rhinoplasty; in individuals whose educational level is higher than a high school diploma, there was a higher level of satisfaction in terms of nasal hump, the display of the middle-lower nose, proportion of the nose compared to the face, and the general satisfaction.

In the present study, the opinions of patients and surgeons were investigated in the first month as well as the third month after rhinoplasty; the findings indicated that the satisfaction level of both patients and surgeons was higher in the third month after rhinoplasty.

The satisfaction level of patients and surgeons were significantly different in terms of nostrils, upward sloping of the nose, and the display of the middle-lower nose. The surgeons’ satisfaction with upward-sloping of the nose and the display of the middle-lower nose was higher than that of the patients. In terms of other investigated factors, the satisfaction level of patients and surgeons was not significant.

## Conclusion

In the present study, given the limitations, including the lack of referral and similar cases, the researchers failed to compare some of the cases. However, it is recommended to use more samples in future studies. Moreover, the authors recommend investigating the satisfaction level several times, as well as longer time intervals.

In our study, it was proven that variables such as age, educational level, gender, and marital status could affect the patients’ expectations and satisfaction. The more time passes from rhinoplasty, the higher the level of satisfaction with nasal beauty and proportions of the patients and surgeons. The satisfaction level of patients and surgeons with nasal beauty and proportions was similar in most cases.

## Conflict of Interest

The authors confirm that there are no conflicts of interest.

## References

[1] Freiberg A, Giguere D, Ross DC, Taylor JR, Bell T, Kerluke LD (1997). Are patients satisfied with results from residents performing aesthetic surgery?. Plastic and cosmetic surgery.

[2] Abbasi H, Sharifi R, Mozaffari HR, Rezaei F, Aghai A, Akbari S (2016). Quality of life before and after rhrinoplasty among iranian patients. International journal of tropical medicine.

[3] D D, W S, D S, A S (1988). Otolaryngology Head and neck Surgery.

[4] Roe JO (1970). The Deformity Termed “PUG Nose” And Its Correction, By A Simple Operation.e. Plastic and cosmetic surgery.

[5] Dieffenbach J (1845). Die Operative Chirurgie.

[6] Saleh AM, Younes A, Friedman O (2012). Cosmetics and function: quality-of-life changes after rhinoplasty surgery. The Laryngoscope.

[7] Ching S, Thoma A, McCabe RE, Antony MM (2003). Measuring outcomes in aesthetic surgery: a comprehensive review of the literature. Plastic and cosmetic surgery.

[8] Guyuron B, Bokhari F (1996). Patient satisfaction following rhinoplasty. Aesthetic Plastic Surgery.

[9] Mckinney P, Cock J (1981). A Critical Evaluation of 200 Rhinoplasties. Annals of Plastic Surgery.

[10] Meyer L, Jacobsson S (1986). The predictive validity of psychosocial factors for patients’ acceptance of rhinoplasty. Ann Plast Surg.

[11] Grossbart TA, Sarwer DB (1999). Cosmetic surgery: Surgical tools—Psychosocial goals. Seminars in cutaneous medicine and surgery.

[12] Sena Esteves S, Gonçalves Ferreira M, Carvalho Almeida J, Abrunhosa J, Almeida e Sousa C (2017). Evaluation of aesthetic and functional outcomes in rhinoplasty surgery: a prospective study. Braz J Otorhinolaryngol.

[13] Rod R (2001). Mirror, Mirror on the Wall: When the Postoperative Reflection Does Not Meet Patients’ Expectations Plastic & Cosmetic Surgery.

[14] Yu K, Kim A, Pearlman SJ (2010). Functional and aesthetic concerns of patients seeking revision rhinoplasty. Archives of facial plastic surgery.

[15] Rodman R, Kridel R (2016). A Staging System for Revision Rhinoplasty: A Review. JAMA facial plastic surgery.

[16] Swami V (2009). Body appreciation, media influence, and weight status predict consideration of cosmetic surgery among female undergraduates. Body image.

[17] Litner JA, Rotenberg BW, Dennis M, Adamson PA (2008). Impact of cosmetic facial surgery on satisfaction with appearance and quality of life. Archives of facial plastic surgery.

